# Wonders of the Sunlight

**Published:** 1886-06

**Authors:** E. D. Babbitt

**Affiliations:** 20 University Place, New York


### WONDERS OF THE SUNLIGHT.

BY E. D. BABBITT, M. D.

The object of this article is not to describe the marvels and the vastness of the sun itself, or to enlarge upon the amazing velocity of 186,000 miles a second with which light sweeps through the heavens, but to show its immensely vitalizing and purifying power in connection with human life. I will commence with the remark that clothing, especially that which is dark, absorbs the light and prevents it from reaching the body with its enkindling power. First let us notice the great strength and vitality of those races who go partly or wholly nude in the air and sunlight.

The Andamaners or Mincopies are a race of people who go entirely naked and occupy some islands east of India. These people have wonderful strength and health. "Capt. Mouatt got up several races between the Mincopies and their own prize crew in their favorite boat. In point of fact there was never any race at all, the Andamaners having it all their own way and winning as they liked...... The sailors were hopelessly beaten, although they strained themselves so much that they felt the results for some time afterward.—*Uncivilized Races of Men, by Rev. J. G. Wood, M. A., Vol. II, p.* 214.

The Dyaks of Borneo wear nothing but a simple strip of cloth called a chawat around the loins. They are able to walk the strongest Europeans down and then carry them by the mile on their backs. They walk off for hours with hundreds of pounds, as though it was but a trifling matter. Mr. Hornaday, a traveler who spent two years among them, pronounced them much more chaste and moral than any of the so-called civilized races.

Speaking of the California Indians Mr. Robt. Brown says: "When not afflicted with hereditary disease they are long-lived, many having died with the reputation of being more than 120 years old. They are said never to catch cold, though often going about in cold winters almost naked.—*Races of Mankind, Vol. I, p.* 162.

It will perhaps impress the lesson I would teach upon the reader all the better by some comparisons of races who dress and who do not. Thus the Eskemos on one hand, who live in a very cold climate, bundle up very tightly with furs, and average only about half the strength of an ordinary white man. On the other hand, the inhabitants of Terra del Fuego, which is south of South America, and so cold that a European has frozen to death in the Summer with his clothing on, can survive from age to age with no clothing excepting a certain amount of grease and dirt on their skin. The women plunge into the icy water after fish, and from their abundant exercise become as strong as the men. The men are twice as strong as Europeans, or about four times as strong as the Eskemos.

A still more striking comparison is that between two similar races, the Chinese, who wear clothing, and the Japanese, a large body of whom spend much of their time nude or nearly so. The Chinese are below the average size and are not very strong or very brave. The Japanese are larger, more powerful, braver, more virtuous, more intellectual, and possessing a tide of vitality so marvelous as to enable them to endure a degree of heat or cold which would kill people who wear clothing. Mrs. Brassey, the English traveler, saw seventy of them entirely nude in a quarry on a day when the cold was far below freezing point.

The Kaffirs of southeastern Africa generally wear no clothing and are famous for their magnificent developement, equal to the finest Grecian statuary. Dr. F. G. Welch, a prominent physician of New

York who spent some time in their midst, often saw them throw bags weighing 600 pounds upon their heads and carry them off, and in one case saw a man hoist 1200 pounds upon his head. Some of them would run 90 or 100 miles ou a stretch.

Other examples could be adduced to show that in communities where the bodies of the people are exposed to the great vitalizing force of sunlight, as well as to the atmospheric electricities, they possess on an average from two to four times the power and endurance cf those who are closely wrapped about with clothing. It would be well now to inquire into the health of of these people, although the fact of their having such a superb strength is of itself a proof of superior health. The African traveler, Sir Samuel W. Baker, says that Livingstone is correct in declaring that hydrophobia does not exist in the tropics, and that idiocy and insanity are rarely seen in Central African countries. Speaking of the Bakwains, who go nearly nude, he says : "The diseases known among them are remarkably few. There is no consumption or scrofula, and insanity and hydrocephalus are rare. Cancer and cholera are quite unknown...... A certain loathsome disease dies out in the interior of Africa without medicine." The same disease was unknown in Japan until introduced by Europeans, and Livingstone makes the same remark about Africa, which, however, when thus taken, is soon expelled from the system by the sun-exposed condition of the people. The native tribes of men and women in Guiana go entirely nude. "As is the case with most uncivilized nations," says the Rev. J. G. Wood, "the Guinian mothers think but little of the event which lays a civilized European on a bed of sickness for weeks." Mr. Brett saw one Waran woman, only two hours after the birth of her child, tie up her hammock and carry it together with her newly-born infant, from one house to another. Compare such people with ourselves, and especially with the Eskemos, who shut out heaven's light and air from their bodies more thoroughly perhaps than any other people on earth. There "after a child is born the mother is obliged to confine herself to her own igloo for some months."

Such is the strength and almost entire immunity from disease of those who live near to nature and who have no ponderous volumes on materia medica. Contrast with these the people who live in shaded rooms with bodies closely wadded about with clothing, and we shall find among the latter pale, cadaverous features and some serious ailment in almost every family.

The monstrosities of Cretinism come wholly from living in deep valleys between mountains where the direct light never comes. The people often grow up blind or deaf or idiotic or deformed, and their scrofulous systems develope immense goitres. Sir James Wylie, late physician to the Emperor of Russia, found that there were four times the disease and death on the shaded side of hospitals that there was on the sunny side, and this discovery led to a reform in the whole hospital system of Russia.

But how are we to appropriate this celestial medicine of which we are in such dying need and use it over the whole surface of the body? We cannot adopt the undress *costume* of what are called the uncivilized races, and many of us live in cities or thickly settled communities where we cannot roam out of doors in natures unadorned simplicity. I will see if I cannot, in part at least, remove this difficulty and lead my readers into the pathway of power.

One method is to go into a warm room where the sun shines, shove the foot of a lounge or cot into the light next the window, the head being toward the middle of the room; then undress and lie down on the back with the sun on the whole front up to the cheeks, but have the eyes and upper head in the shadow. Lie thus from thirty to sixty minutes, and then turn over and let the sun come on the back equally long. If the skin is florid and liable to burn too much, spread some blue veiling over the body. When the spine is exposed, if it is hot or irritable, spread two or three thicknesses of the same veiling over it and it will prove very curative. Sunstrokes come principally from heating the upper brain. This sunning of the body will tend to relieve the brain. These baths continued once a day, when the sun shines (for weeks) will give a great addition to the vitality, toughness and cheer of the system.

A much more powerful and effective sun bath can be taken inside of a kind of portable solarium, which has large reflectors to throw a great amount of light on the body and glass panes of different colors to rest over any diseased part and affect it according to its condition. When the instrument is shut up and the body enclosed, the head being outside in the cool air, a very fine perspiration starts and a great purifying as well as vitalizing process takes place. Luminous heat is far more refined and upbuilding than the ordinary dead heat, and if I should enumerate some of the cures of consumption, dropsy, scrofula, rheumatism, sciatica, etc. that have been made by this kind of solar bath, they would be considered almost too marvelous for belief.

"Twelve years ago," said Walt Whitman, "I came to Camden to die. But every day I went into the country and naked bathed in the sunshine, lived with the birds and the squirrels, and played in the water with the fishes. I recovered my health from nature."

I will now suggest a *sun play-ground* for children, which in a single week in summer will bring to them a new life, strength and toughness. Build an enclosure twelve or more feet square and place one or two loads of sand therein. Then when the sun is shining shut the children in there and let them play, nearly if not quite naked. The sand will feel delightful to their bare skin, and they will roll over in it, as well as jump and keel and bury their legs in its soft depths. While the sand will thus act chemically to absorb certain impurities from the system, the air and sunlight will give them a new power. The principal objection to this solar pen, if carried out quite generally, is that the doctors might suffer from a lack of business.

To sum up the points relating to this subject we may say :

1st—The races of men who receive the sunlight and air to their bodies from wearing little or no clothing, have far greater strength, immunity from disease, and power of recuperation when wounded, than have those nations that exclude these forces by means of heavy clothing, however skillful the latter may be in medical science. This heavy clothing not only weakens, but developes grossness and animalism by confining the vital heat to the body and shutting out the purifying influences of nature. As proof of this it is well known that the nude races, such as the Kaffirs, the Dyaks, the New Guinians and the Japanese are decidedly more chaste than the people of Europe or America. This shows that the human body itself is not necessarily impure, being the workmanship of the Divine Hand.

2d—Clothing may be worn in moderation, especially if it is sufficiently loose to allow free circulation, and sufficiently thin and light-colored in summer to admit the light to the skin and yet permit a powerful physical development in case the body is vitalized and purified by means of solar baths and solar sweat-baths.

3d—In case persons live in shadow, the mental forces sink into ruin, health is destroyed and the features lose their beauty of form and color.

4th—The sunlight resting upon the skin develops the most exquisite counter irritation over the whole body, drawing internal congestions outward, destroying poisonous germs in the blood and preventing morbid conditions.

5th—Considering that the sun is admitted to be the mightiest power in nature and the great up-builder of animal and vegetable life, does it not look almost like insanity, as well as ignorance, for us to neglect the application of its divine potency to our bodies?

(20 University Place, New York.)

